# Assessing the association between diet quality and sociodemographic factors in young Saudi adults

**DOI:** 10.3389/fnut.2025.1641284

**Published:** 2025-07-21

**Authors:** Abeer Salman Alzaben, Kholoud Rashed Alresheedi, Huny M. Bakry, Rahaf Abdullah Ozayb, Halah Abdulaziz Aldawsari, Arwa Obaid Alnamshan, Nahla M. Bawazeer

**Affiliations:** Department of Health Sciences, College of Health and Rehabilitation Sciences, Princess Nourah bint Abdulrahman University, Riyadh, Saudi Arabia

**Keywords:** diet quality, short-healthy eating index, sociodemographic, sHEI, Arabic

## Abstract

**Objective:**

This study aimed to evaluate diet quality (DQ) using the Short Healthy Eating Index (sHEI) and explore the relationship between DQ and sociodemographic factors among young adults in Saudi Arabia.

**Methods:**

This observational cross-sectional study was conducted in Saudi Arabia among young adults aged 18–25 years, through a questionnaire distributed online using social media. The participants provided consent and demographic information, and DQ was assessed using a validated sHEI questionnaire. The questionnaire was translated to Arabic and adapted for local relevance Data analyses were performed. Statistical significance was defined as *p* ≤ 0.05.

**Results:**

A total of 605 participants (average age 20.8 ± 2.0 years old) were recruited. More than half of the participants had a normal body mass index. The average total sHEI score, adequacy, and moderation scores were 44.98 ± 9.36, 29.56 ± 7.16, and 15.40 ± 5.71, respectively. BMI was negatively associated (weak association) with the adequacy score (*p* < 0.05). There were significant associations between the adequacy score, total sHEI score, sex, and region (*p* < 0.05). Being female was associated with good adequacy scores (*p* = 0.010). Being female (*p* = 0.001) and residing in the southern region area (*p* = 0.028) were associated with good total sHEI scores (*p* = 0.005).

**Conclusion:**

Most individuals had a low sHEI, indicating poor DQ. Nutrition education should focus on DQ, sustainable nutrition, and eating behaviors. Future studies should assess the association between DQ and sociodemographic factors such as gender region and other lifestyle factors such as physical activity, sleep pattern, and smoking.

## Introduction

Diet quality (DQ) is crucial for food security and a vital metric in nutrition, wellness, and preventive programs because of its association with chronic illnesses (1). Changing diets, increasing malnutrition concerns, and measuring DQ instead of focusing on energy sufficiency or single nutrient is gaining momentum. A high DQ is linked to a lower risk of chronic diseases such as obesity, type 2 diabetes, hypertension, and cardiovascular disease, serving as a protective factor. Conversely, low DQ is associated with a higher risk of obesity and chronic diseases (2).

A balanced diet is crucial for health, and meets various nutritional needs (3). DQ measures the variety and quality of food according to dietary guidelines (4). These guidelines recommend a high-quality diet rich in vegetables, fruits, seeds, legumes, and whole grains while limiting sodium, saturated fats, and sugars, indicating a low-quality diet (5). In Saudi Arabia, dietary habits include high consumption of fats, salt, and sugar, with a low intake of vegetables, fruits, dairy, nuts, and fish, raising concerns about DQ (6, 7). This unhealthy pattern is prevalent among Saudi adolescents, who often consume high-energy foods and beverages while skipping nutritious meals (8). Additionally, recent research has indicated that female university students fail to meet the recommended intake of vegetables and fruits (4).

Several tools are available to validate and measure overall DQ in the global population. The Healthy Eating Index (HEI) is one of the oldest tools used to assess dietary adequacy. It evaluates the intake of nutrient adequacy; in addition, it estimates the overall balance of the diet and energy intake (9). HEI was evaluated and compared with the US Dietary Guidelines (10). HEI have undergone major developments and may be useful for intervention studies (9). Therefore, experts developed the Short Healthy Eating Index (sHEI) tool for assessing DQ, which is easy to use for both respondents and researchers. It is a 22-item tool that measures consumption in terms of nutrients and quantity; it includes (fruits, vegetables, dairy, added sugar, sugar from sugar-sweetened beverages, and calcium) (2).

Although DQ is a critical measure of overall diet and health status, to the best of our knowledge, no study has assessed DQ using the sHEI among young adults of both sexes in Saudi Arabia. Young adulthood is recognized as a critical developmental stage wherein long-term dietary habits and lifestyle behaviors are established. It is accompanied by major transitions including increased autonomy, changes in living arrangements, academic or occupational demands, and exposure to new social environments. These factors collectively contribute to a heightened risk of adopting unhealthy dietary patterns and sedentary behaviors, which may persist into later adulthood and influence long-term health outcomes. Therefore, this study aimed to evaluate the DQ using the sHEI and explore the relationship between the sHEI and sociodemographic factors among young female and male adults in Saudi Arabia. This study provides valuable insights into dietary habits and guides public health efforts to enhance DQ, prevent diet-related chronic diseases, and promote nutritional wellbeing.

## Materials and methods

### Participants and sample size

This study was approved by the Institutional Review Board committee (IRB# Exempt Approval 22–0935 dated 27 Nov 2022). A detailed consent form was given to all participants that included data confidentiality and voluntary participation information. The sample size was estimated based on a population of 4–800 individuals aged 18–25 years by 2022 (11). The sample size calculation was conducted assuming a prevalence of 50% (*p* ≤ 0.05) due to the lack of established information regarding the prevalence of DQ. The final sample size was 384. However, 605 participants were recruited to increase the statistical power, representativeness and generalizability of the results.

This cross-sectional study included young adults aged 18–25 years, living in Saudi Arabia. The exclusion criteria included pregnant or lactating females or people with chronic diseases that alters food behaviors (such as food allergies, celiac disease, and diabetes mellitus). An online questionnaire was distributed in Arabic through social media platforms (WhatsApp, Twitter, and Telegram), using snowball sampling.

### Sociodemographic characteristics

Sociodemographic characteristics were collected by asking participants to provide specific demographic information including age, sex, education level, nationality, occupation, marital status, and region of residence in Saudi Arabia. Additionally, participants were asked to record their weight (kg) and height (cm) to calculate body mass index (BMI) (kg/m^2^). BMI was categorized into three groups: underweight, healthy or normal, and overweight or obese (12).

### Diet quality

Diet Quality was assessed and validated using sHEI. The sHEI is a 22-item tool and was developed and validated in the college population by Colby et al., 2020 (2). The sHEI scoring system depends on 13 components (total fruits, whole fruits, total vegetables, greens and beans, whole grains, dairy, total protein, seafood, plant protein, fatty acids, refined grains, sodium, added sugars, and saturated fats), based on the dietary guidelines (2, 13, 14). Recently, the sHEI was translated into Arabic and its reliability and validity were assessed in a young Saudi adult population (15). The total score was calculated as the sum of the 13 components. The adequacy score reflects food components that should be consumed in sufficient quantities for a healthy diet. The adequacy score was calculated as the sum of nine components (total fruits, whole fruits, total vegetables, greens and beans, whole grains, dairy, total protein, seafood and plant protein, and fatty acids) (16). The moderation score reflects the food components that should be consumed in limited amounts to avoid negative health impacts. The moderation score was calculated as the sum of four components (refined grains, sodium, added sugars, and saturated fats) (16). The cutoff points for good and poor DQ scores were above and below the median, respectively.

### Statistical analysis

The Statistical Package for the Social Sciences (SPSS) V.26 software was used. The cut-off point for significance was *p*-value ≤ 0.05. Categorical variables are expressed as numbers (n) and percentages (%), whereas continuous variables are expressed as mean and standard deviation (SD). To test the association between DQ and sociodemographic characteristics, a chi-square test was conducted between two categorical variables: sociodemographic characteristics (sex, BMI, region, marital status, and education) and sHEI (poor vs. good). After performing chi square, *post hoc* analysis was performed to pinpoint region of significance. Spearman’s correlation was conducted between sHEI (adequacy, moderation, and total score), age, and BMI. A multinomial logistic regression was conducted between the sHEI (adequacy, moderation, and total scores) and sociodemographic characteristics.

## Results

### Sociodemographic characteristics

Table 1 presents the participants’ general characteristics. A total of 605 participants were included in the analysis: 89.4% were females, and 10.6% were males. The age varied between 18 to 25 years. More than half of the participants had a normal BMI (56.5%). Most of the participants lived in the middle region of Saudi Arabia (58.3%); had a diploma, university, or postgraduate level of education (78.5%); and were unmarried (94.2%).

**Table 1:** Sociodemographic characteristics of participants (*n* = 605).

Variable	Mean ± SD or No. (%)
Age (years)	20.8 ± 2.0
BMI (kg/m^2^)	22.61 ± 5.25
Gender	
Female	541 (89.4%)
Male	64 (10.6%)
Region	
Eastern region	48 (7.9%)
Southern region	50 (8.3%)
Middle region	353 (58.3%)
Northern region	35 (5.8%)
Western region	119 (19.7%)
Level of education	
High school or less	130 (21.5%)
Diploma/University/Postgraduate	475 (78.5%)
Social status	
Unmarried	570 (94.2%)
Married	35 (5.8%)
BMI category	
Underweight	114 (18.8%)
Normal	342 (56.5%)
Overweight/Obese	149 (24.6%)

### Diet quality

**Figure 1:**
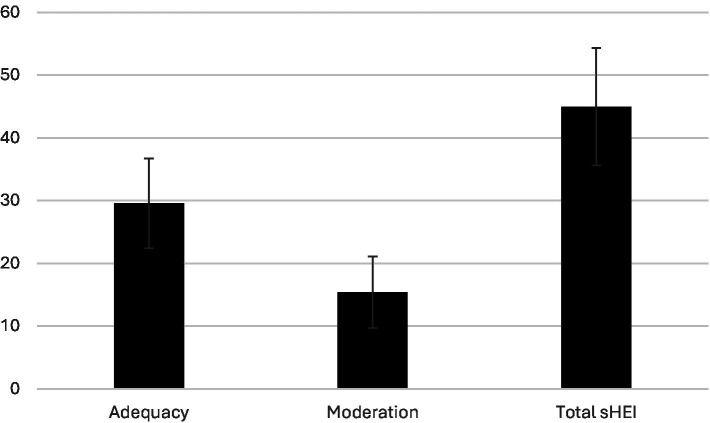
The average adequacy, moderation and total sHEI scores of participants (*n* = 605).

Figure 1 shows the participants’ total sHEI, adequacy, and moderation scores (*n* = 605). The average total sHEI score was 44.98 ± 9.36 (range 23.42–73.61). The mean adequacy score was 29.56 ± 7.16 (range 14.53–45.09). The mean moderation score was 15.40 ± 5.71 (range 4.65–28.52). Table 2 shows the sHEI component scores (*n* = 605). Whole fruit consumption was lowest score in the sHEI group.

**Table 2:** The sHEI component scores of participants (*n* = 605).

sHEI components	Items	Mean	SD	Min	Max
Adequacy	Total fruits	2.15	2.046	0	5
	Whole fruits	1.72	1.984	0	5
	Total vegetables	2.5	0.767	1.6	3.56
	Greens and beans	3.91	2.061	0	5
	Whole grains	4.10	2.343	0.51	6.94
	Dairy	4.53	1.245	3.22	6.51
	Total protein	4.91	0.206	4.11	4.97
	Seafood and plant protein	2.25	1.319	0.49	4.20
	Fatty acids	3.45	1.448	2.56	5.93
Moderation	Refined grains	4.04	2.501	2.13	9.25
	Sodium	5.01	1.101	0.70	6.07
	Added Sugars	3.29	3.558	0	10
	Saturated Fats	3.04	1.279	1.82	4.64

### Association between general characteristics and diet quality

Table 3 shows the associations between adequacy, moderation, total sHEI scores, and sociodemographic characteristics. Sex and region were significantly associated with adequacy and total sHEI scores (*p* < 0.05). After performing chi square to compare regions regarding adequacy score and total sHEI score, *post hoc* analysis was performed to pinpoint region of significance. *Post hoc* analysis shows that southern rejoin were associated with adequacy and total sHEI score (*p* = 0.006). Table 4 shows the Spearman’s correlation between adequacy, moderation, total sHEI scores, age, and BMI. BMI was negatively associated (weakly associated) with the adequacy score (*p* < 0.05). Table 5 presents the results of the multinomial logistic regression analysis of adequacy, moderation, total sHEI scores, and sociodemographic characteristics. Being female was associated with good adequacy scores (*p* = 0.010). Being female (*p* = 0.001) and residing in the southern rejoint area (*p* = 0.028) were associated with good total sHEI scores (*p* = 0.005).

**Table 3:** The association between adequacy, moderation and total sHEI score and sociodemographic characteristics of participants (*n* = 605).

Variables	Adequacy score				Moderation score				Total sHEI score			
	Poor	Good	Chi-Square	*p*-value	Poor	Good	Chi-Square	*p*-value	Poor	Good	Chi-Square	*p*-value
Gender												
Male	43 (76.2%)	21 (32.8%)	8.37	0.004	35 (54.7%)	29 (45.3%)	1.876	0.171	45 (70.3%)	19 (29.7%)	11.7	0.001
Female	260 (48.1%)	281 (51.9%)			247 (45.7%)	294 (54.3%)			258 (47.7%)	283 (52.3%)		
BMI categories												
Normal	156 (45.7%)	185 (54.3%)	5.77	0.055	152 (44.6%)	189 (55.4%)	1.8	0.39	156 (45.7%)	185 (54.3%)	5.7	0.058
Under wt	62 (54.4%)	52 (45.6%)			59 (51.8%)	55 (84.2%)			64 (56.1%)	50 (43.9%)		
Over wt /Obese	84 (56.4%)	65 (43.6%)			71 (47.7%)	78 (52.3%)			82 (55%)	67 (45%)		
Region												
Middle	191 (54.1%)	162 (45.9%)	10.3	0.040	159 (45%)	194 (55%)	3.279	0.512	193 (54.7%)	160 (54.3%)	11.023	0.026
Northen	18 (51.4%)	17 (48.6%)			18 (51.4%)	17 (48.6%)			18 (51.4%)	17 (48.6%)		
Southern*	16 (32%)	34 (68%)			27 (54%)	23 (46%)			16 (32%)	34 (68%)		
Eastern	20 (41.7%)	28 (58.3%)			26 (54.2%)	22 (45.8%)			21 (43.7%)	27 (56.3%)		
Western	58 (48.7%)	61 (51.3%)			52 (43.7%)	67 (56.3%)			55 (46.2%)	64 (53.8%)		
Marital status												
Married	21 (60%)	14 (40%)	1.462	0.227	18 (51.4%)	17 (48.6%)	0.346	0.556	17 (48.6%)	18 (51.4%)	0.034	0.854
Unmarried	282 (49.5%)	288 (50.5%)			264 (46.3%)	306 (53.7%)			286 (50.2%)	284 (49.8%)		
Educational level												
High school or less	63 (48.5%)	67 (51.5%)	0.1	0.75	57 (43.8%)	73 (56.2%)	0.377	0.53	63 (48.5%)	67 (51.5%)	0.1	0.75
Diploma/University/Postgraduate	240 (50.5%)	235 (49.5%)			225 (47.4%)	250 (52.6%)			240 (50.5%)	235 (49.5%)		

*Post hoc p-value = 0.006 in adequacy and total sHEI score. Data is presented as number of participants (No.) and percentage (%). wt, weight.

**Table 4:** The Spearman correlation between adequacy, moderation and total sHEI score and age and BMI of participants (*n* = 605).

Variables		Adequacy	Moderation	Total sHEI
Age	Correlation	−0.076	0.070	−0.018
	*p*-value	0.063	0.084	0.661
BMI	Correlation	−0.107	0.023	−0.062
	*p*-value	0.008	0.575	0.130

**Table 5:** The multinomial logistic regression of adequacy, moderation and total sHEI score and general characteristics of participants (*n* = 605).

Variables	Good Adequacy score^*^				Good Moderation score^**^				Good Total sHEI score^***^			
	−2 Log Likelihood of reduced model	Chi-Square	df	*p*-value	−2 Log Likelihood of reduced model	Chi-Square	df	*p*-value	−2 Log Likelihood of reduced model	Chi-Square	df	*p*-value
Intercept	815.750	0.000	0		822.294	0.000	0		809.705	0.000	0	
Age	816.039	0.290	1	0.590	827.002	4.708	1	0.030	810.080	0.375	1	0.541
BMI	816.715	0.965	1	0.326	822.346	0.053	1	0.819	809.913	0.208	1	0.648
Region (southern)	825.208	9.458	4	0.049	824.931	2.637	4	0.620	820.614	10.909	4	0.028
Education	815.824	0.074	1	0.785	823.938	1.644	1	0.200	809.723	0.018	1	0.893
Marital Status	816.477	0.728	1	0.394	823.824	1.531	1	0.216	809.716	0.011	1	0.916
Gender (female)	822.452	6.702	1	0.010	825.564	3.270	1	0.071	821.135	11.430	1	0.001

*p-value of Adequacy model is 0.01. **p-value of moderation model is 0.285. ***p-value of total model is 0.005.

## Discussion

Several methodologies have been developed and used to assess overall DQ in different populations worldwide one of the earliest DQ instrument is the HEI, that measures adequacy, moderation, and overall DQ (2, 15). This study aimed to measure DQ using sHEI and explore the relationship between sHEI and sociodemographic factors among a sample of young adults in Saudi Arabia. The average sHEi score among the samples was 45, indicating a poor DQ. This result is consistent with a recent study conducted among Saudi colleges in which the average sHEI was 43 (17). Another study assessed global and regional DQ using the Alternative Healthy Eating Index (AHEI) and reported that the AHEI score in the Middle East/North African region ranged from 30 to 40 out of 100 (18). This is considered a poor DQ. However, the study examined national changes in AHEI scores between 1990 and 2018 (18). They concluded that DQ improved between 1990 and 2018 in the Middle East/North African region (18). In addition, the study examined DQ in children and adults, and the DQ did not differ between adults and children. If children have poor DQ early in life, they are more likely to maintain poor dietary habits as they grow older (19).

DQ is influenced by various factors including age, sex, body mass index (BMI), culture, physical activity, smoking status, socioeconomic status, race, ethnicity, education level, and country (20, 21). One study reported that females had better DQ than males, and children and older adults had better DQ than younger and middle-aged adults (20). Furthermore, socioeconomic status affects DQ. Lower socioeconomic status is associated with poor DQ and nutritional value, whereas higher status is associated with good DQ (22). In addition, DQ improved with education level. Higher education typically leads to better dietary knowledge than lower education (23).

Several studies have provided evidence of unhealthy dietary patterns among adults, adolescents, and children in Saudi Arabia (24–30). Studies have reported high consumption of soft drinks among adults in Saudi Arabia (24, 25). Another study showed a very high consumption of added sugar (73 g of added sugar per day), with the highest consumption in the northern region, followed by the Southern, Western, Central, and Eastern regions (26). Another study reported lower intakes of fruits, vegetables, and fish among in Saudi adults (29). Saudi adults consume approximately six servings/week of fruits and vegetables and one serving/week of each fish (29). A randomized controlled trial reported low consumption of fruits and vegetables among Saudi Adolescents consistent with another study in Saudi females, which reported that 97% of Saudi females consumed one–three fruit and vegetable servings per day (27, 28).

The current study observed significant regional differences in the adequacy and total sHEI scores. Several studies have reported regional differences in eating habits, diet quality and lifestyle factors which may be explained by differences in cultural norms, socioeconomic conditions, and accessibility (26, 31, 32). This leads to notable regional variations in the prevalence of overweight and obesity among Saudi children and adolescents, suggesting that environmental and behavioral factors vary considerably across geographic locations (31). A randomized controlled trial reported that an increase in DQ using the AHEI-2010 was associated with a reduction in body weight and fat mass in overweight adults (33). Individuals with normal body weight were expected to adhere to a healthier DQ.

Studies have indicated sex as a factor influencing DQ (17, 18, 20, 34, 35). A study reported that females presented with 6 points higher HEI than males (17). Similarly, another global study reported females had higher AHEI scores due to healthier dietary intake and dietary patterns (18). Also, 69% of males and 57% of females exceeded their saturated fat intake as per another study (36). While most females (approximately 86%) met the RDA for dietary fiber, only 67% of males met the RDA for dietary fiber (36). Likewise, females also have a higher intake of vitamins A and C (36). The current study observed that females had better DQ than males in adequacy score and total score. Although, the male sample size mandates caution.

In addition, other lifestyle factors may affect diet quality (social media use, sleep pattern, exercise, smoking, and alcohol) (30, 37). Current evidence indicates a positive association between physical activity, sufficient sleep, and diet quality (38–41). Additionally, the use of tobacco and alcohol is linked to negative dietary outcomes (42, 43). Several strengths of this study need to be reported, including the use of a previously valid and reliable tool to assess DQ. The sHEI was previously translated into Arabic and validated in young Arabs (15). The analysis also considered potential confounders. Finally, the sample size calculations indicated that the study size was appropriate.

## Study limitations

Despite adding knowledge, the study has a few limitations. This study employed a snowball sampling method, which carries inherent limitations. Thus, the generalizability may be limited. Future studies are encouraged to adopt probability-based sampling techniques to ensure representativeness and external validity. Furthermore, the proportion of males was lower than females which may affect the generalizability of the findings. In addition, the use of self-reported questionnaire and BMI metric.

## Conclusion

A poor DQ was observed in a majority of the sample. To address this issue, nutrition education initiatives should specifically target behaviors such as increasing fruit and vegetable intake, reducing consumption of processed and high-sugar foods, and promoting balanced, sustainable eating patterns. These interventions should be implemented in schools, universities, workplaces, and primary healthcare settings to reach different age and social groups effectively. Given the observed gender and regional disparities in diet quality region-specific strategies and gender-responsive interventions are crucial. For instance, tailored programs for females and populations in underrepresented regions can help address specific dietary challenges. The current findings underscore the importance of increasing public health awareness about healthy eating. Nutrition education initiatives tailored to meet context sensitive needs targeting food intake behaviors through educational and work institutes should be promoted to control poor DQ. Finally, future studies should adopt probability-based sampling methods to assess the association between DQ and sociodemographic factors such as gender region and other lifestyle factors such as physical activity, sleep pattern, and smoking to ensure more representative data and improve the generalizability of findings.

## Data Availability

The raw data supporting the conclusions of this article will be made available by the authors, without undue reservation.
